# Learning from serum markers reflecting endothelial activation: longitudinal data in childhood-onset systemic lupus erythematosus

**DOI:** 10.1136/lupus-2024-001190

**Published:** 2024-09-05

**Authors:** Sandy C Bergkamp, Nick D Bergkamp, Mohamed Javad Wahadat, Mariken P Gruppen, Amara Nassar-Sheikh Rashid, Sander W Tas, Martine J Smit, Marjan A Versnel, J Merlijn van den Berg, Sylvia Kamphuis, Dieneke Schonenberg-Meinema

**Affiliations:** 1 Department of Paediatric Immunology, Rheumatology and Infectious Diseases, Emma Children’s Hospital, Amsterdam University Medical Centres (AUMC), University of Amsterdam, Amsterdam, The Netherlands; 2 Amsterdam Institute for Molecular and Life Sciences (AIMMS), Division of Medicinal Chemistry, Faculty of Science, Vrije Universiteit Amsterdam, Amsterdam, The Netherlands; 3 Department of Paediatric Rheumatology, Sophia Children’s Hospital, Erasmus University Medical Centre, Rotterdam, The Netherlands; 4 Department of Immunology, Erasmus Medical Centre, Rotterdam, The Netherlands; 5 Department of Paediatrics, Zaans Medisch Centrum, Zaandam, The Netherlands; 6 Amsterdam Rheumatology and Immunology Centre, Department of Rheumatology and Clinical Immunology, and Laboratory for Experimental Immunology, Amsterdam University Medical Centres (AUMC), University of Amsterdam, Amsterdam, The Netherlands

**Keywords:** Autoimmune Diseases, Cardiovascular Diseases, Lupus Erythematosus, Systemic, Lipids

## Abstract

**Objectives:**

In childhood-onset SLE (cSLE), patients have an increased risk of premature atherosclerosis. The pathophysiological mechanisms for this premature atherosclerosis are not yet completely understood, but besides traditional risk factors, the endothelium plays a major role. The first aim of this study was to measure levels of SLE-associated markers involved in endothelial cell (EC) function and lipids in a cSLE cohort longitudinally in comparison with healthy controls (HC). Next aim was to correlate these levels with Systemic Lupus Erythematosus Disease Activity Index (SLEDAI) and nailfold capillaroscopic patterns.

**Methods:**

Blood serum samples, videocapillaroscopy images and patient characteristics were collected in a multicentre longitudinal cSLE cohort and from age and sex comparable HC. Disease activity was evaluated by SLEDAI. A total of 15 EC markers and six lipids were measured in two longitudinal cSLE samples (minimum interval of 6 months) and in HC. Nailfold videocapillaroscopy images were scored according to the guidelines from the EULAR Study Group on Microcirculation in Rheumatic Diseases.

**Results:**

In total, 47 patients with cSLE and 42 HCs were analysed. Median age at diagnosis was 15 years (IQR 12–16 years). Median time between t=1 and t=2 was 14.5 months (IQR 9–24 months). Median SLEDAI was 12 (IQR 6–18) at t=1 and 2 (IQR 1–4) at t=2. Serum levels of angiopoietin-2, CCL2, CXCL10, GAS6, pentraxin-3, thrombomodulin, VCAM-1 and vWF-A2 were elevated in cSLE compared with HC at t=1. While many elevated EC markers at t=1 normalised over time after treatment, several markers remained significantly increased compared with HC (angiopoietin-2, CCL2, CXCL10, GAS6, thrombomodulin and VCAM-1).

**Conclusion:**

In serum from patients with cSLE different markers of endothelial activation were dysregulated. While most markers normalised during treatment, others remained elevated in a subset of patients, even during low disease activity. These results suggest a role for the dysregulated endothelium in early and later phases of cSLE, possibly also during lower disease activity.

**Trial registration number:**

NL60885.018.17.

**WHAT IS ALREADY KNOWN ON THIS TOPIC**

**WHAT THIS STUDY ADDS**

- Our study shows that numerous serum markers associated with endothelial cell (EC) function in SLE are upregulated in patients with cSLE compared with healthy controls, particularly in those with untreated disease. We also show that certain EC markers (angiopoietin-2, CCL2, CXCL10, GAS6, thrombomodulin and VCAM-1) could stay persistently upregulated in patients with cSLE, even in states of low disease activity. This suggests that in a subset of patients with cSLE, the endothelium seems to remain in a state of chronic activation irrespective of disease activity.

**HOW THIS STUDY MIGHT AFFECT RESEARCH, PRACTICE OR POLICY**

- We urge for more investigations on the relation between EC dysregulation and increased risk for premature atherosclerosis and cardiovascular disease in SLE. We emphasise that patients with cSLE should be studied as a subgroup because of longer disease duration while being adults.

## Introduction

 Patients suffering from autoimmune diseases have an increased risk of developing atherosclerosis, the most important cause of cardiovascular disease (CVD).^1^ Compared with other autoimmune diseases, patients with SLE have an even higher risk for premature atherosclerosis and the majority of SLE-associated deaths have been attributed to CVD.^2 3^

Childhood-onset SLE (cSLE) represents 10–20% of all SLE cases and is more severe than adult-onset SLE (aSLE), as it is characterised by a more severe disease course and earlier damage accrual.^4^,^7^ cSLE is a life-threatening disease, which results in a 20-fold higher mortality rate in children when compared with healthy peers.^8^ Furthermore, SLE-associated premature atherosclerosis and CVD manifest at a much younger age in cSLE compared with aSLE.^4^ Cardiovascular and cerebrovascular complications have been reported in 5–10% of young adults with cSLE and the majority of these occur between the ages of 20 and 40.^4^

To date, the complex pathophysiological mechanisms underlying premature atherosclerosis in SLE are not completely understood.^9^ Although traditional risk factors (eg, hypertension, obesity and dyslipidaemia) contribute to atherosclerosis and CVD in patients with SLE, non-traditional SLE-specific risk factors (such as corticosteroid use) seem to be just as important.^9^,^11^ A growing body of evidence indicates an important role of endothelial dysfunction in the development of accelerated atherosclerosis in cSLE.^12^,^17^ Different mechanisms play a role, including inflammation triggered by aggregation and oxidation of low-density lipoprotein (LDL), inducing the expression of cell adhesion molecules (CAMs),^18^ activation of endothelial cells (ECs) and higher leucocyte-EC interactions.^19^ Apparently, these events constitute the initial stage of endothelial dysfunction and atherogenesis in SLE.^20 21^ Furthermore, activation of ECs triggers proinflammatory cytokines, for example, CCL2, which play a direct role in accelerated atherosclerosis in SLE.^22^ Neutrophil extracellular traps are a key factor in atherosclerosis in SLE by augmenting EC death and endothelial-to-mesenchymal transition.^23 24^ Lastly, in SLE, endothelial progenitor cells (EPC), which play a crucial role in endothelial repair and vascular homeostasis control, are reduced in number and the function of EPCs is often impaired.^25^ Thus, the endothelium in SLE suffers from inflammation, defective repair and prothrombogenic factors.^25^

To decrease the risk of CVD in cSLE, early-stage detection of atherosclerosis and vascular damage is critical.^26^ Until now, screening protocols for detecting biomarkers that predict those atherosclerotic risks are not routinely implemented in clinical care of patients with SLE (in a possible preventive preatherosclerotic phase). Currently, only investigations are available that detect an atherosclerotic plaque when it is already macroscopically visible with functional microcirculation assessment, such as carotid intima-media thickness test.^15^ Nailfold videocapillaroscopy (NVC) is also a tool to visualise the microvascular pathology. We have shown that the majority of patients with cSLE show an abnormal capillary pattern in nailfolds and that a capillary scleroderma pattern in SLE seems associated with damage.^27 28^ The predominant finding of nailfold microangiopathy in cSLE anatomically looks like capillary leakage and revascularisation and might be due to endothelial dysregulation in SLE which could lead to this microangiopathy.^27^ Following our systematic review and meta-analysis,^29^ we hypothesised that the endothelium in SLE remains dysregulated even in states of low disease activity. If endothelial dysregulation proceeds atherosclerosis, measuring SLE-related markers involved in the EC function in cSLE would give us more insight into the preatherosclerotic phase.

In this study, we measured SLE-related EC markers and serum lipids in cSLE and in healthy controls (HC). We assessed a potential correlation between these EC markers and serum lipids with disease activity and with the nailfold capillary pattern. Moreover, we analysed these EC markers and serum lipids longitudinally.

## Methods

### Study design and patient selection

Between April 2016 and January 2022, patients with cSLE who visited the (out)patient clinics of the Amsterdam UMC and Erasmus MC (Rotterdam) were recruited for a longitudinal prospective cSLE cohort. Inclusion criteria for patients were all patients (new or already diagnosed) with (1) SLE diagnosis according to the 2012 Systemic Lupus International Collaborating Clinics classification criteria and (2) disease diagnosis before the age of 18 years. At diagnosis and during follow-up at study visits every 6–12 months, demographics, autoimmune serology, time of disease onset, disease activity and type of organ involvement were noted. In the majority of the patients, NVC was performed at diagnosis and annually thereafter if possible. Exclusion followed when it was not possible to collect images with good enough quality for analysis (due to thickness of nailfold skin) or when a patient was too sick to undergo capillaroscopy examination. Disease activity was defined by the Systemic Lupus Erythematosus Disease Activity Index 2000 (SLEDAI-2K)^30^; serum samples were collected at study visits and immediately stored at −80°C until analysis.

For this study, selection of data was performed retrospectively on patients from this longitudinal cohort study with preferably two available longitudinal serum samples (minimum interval of 6 months), one with active disease (SLEDAI>4) at t=1 (preferably treatment naïve, at diagnosis) and one, later in time, during low disease activity (SLEDAI≤4) at t=2. To include all available treatment-naïve patients, some of these did not have a sample with low disease activity. Thus, if patients had less than two samples in total, two samples with less than 6 months of interval or two samples with both SLEDAI<4 or both >4, SLEDAI≤4 at t=1, these samples were included for cross-sectional analyses but excluded for the longitudinal analyses.

One-time blood samples from age and sex comparable HCs were obtained from children who were investigated by MRI for joint complaints, and in whom an inflammatory disease had been excluded (JIA-MRI study, NL52625.018.15; approved by the Medical Ethical Committee (MEC) Amsterdam, MEC 2015-046).

### Measurement of serum marker levels and lipids

Based on our previous systematic literature review on (dysregulated) SLE-associated markers involved in EC function,^29^ we selected the following panel of 15 markers (panel 1). Serum levels of CXCL12 (SDF-1), TWEAK and vascular endothelial growth factor (VEGF) were measured using a commercially available ELISA kit (R&D Systems) according to the manufacturer’s instructions. Serum levels of CXCL10 (IP-10), ADAMTS13, angiopoietin-2, pentraxin-3, E-selectin, thrombomodulin, P-selectin, CCL2 (MCP-1), VCAM-1, ICAM-1, vWF-A2 and GAS6 were determined using a tailor-made magnetic bead Luminex Multiplex Assay Kit (R&D Systems), according to manufacturer’s procedures, and measured using a Bioplex 200 system (Bio-Rad). Samples were thawed only once for measurements of marker levels. Technical duplicates of samples were run simultaneously on the same plate, resulting in no need for an extra freeze-thaw cycle.

For those samples with sufficient material left after serum marker-level determinations, lipids were determined (panel 2). Of note, these measurements were assessed in blood that was taken from patients in a non-fasted state. Total cholesterol, high-density lipoprotein-cholesterol (HDL-C), triglyceride (TG) levels, ApoA1 and ApoB were determined by our ISO-certified central laboratory facility. LDL-cholesterol (LDL-C) was calculated by the Friedewald formula. ApoB/ApoA1 was calculated as a ratio. These samples were thawed twice before measurements.

### Nailfold capillaroscopy technique and image analysis

NVC was performed with a ×200 magnification lens from Optilia. In total, eight fingers per patient (excluding the thumbs) were examined. Per finger, four images were stored. Images were collected and evaluated by two investigators (DS-M and SCB with, respectively, 8 and 5 years of experience in performing an analysis of NVC). According to the ‘EULAR Study Group on Microcirculation in Rheumatic Diseases standardised capillaroscopy evaluation chart’, the three different capillary patterns were described. When capillary abnormalities were absent by NVC analysis the capillary pattern was defined as ‘normal’. Microangiopathy was defined as an abnormal capillary pattern (with abnormal capillary morphology and/or capillary haemorrhages), but without criteria for a scleroderma pattern. A capillary scleroderma pattern was defined as extremely lowered density (≤3 capillaries/mm) with abnormal shapes and/or the presence of giant capillaries.^31^ The majority of patients underwent NVC at least once during follow-up. For analysis, the most abnormal capillary pattern ever during follow-up was used.

### Statistical methods

Descriptive statistics were used to analyse demographics of patients with cSLE and HC. Spearman’s rank correlation coefficients were calculated to quantify the relationship between SLEDAI and EC marker levels and lipids. R values between 0.3 and 0.5 were classified as weak relations, between 0.5 and 0.7 as moderate relations and r values >0.7 as strong relations.^32^ Mann-Whitney U tests were used to compare differences between two different groups (for differences between HC and SLE, and differences in HC vs active disease and low disease activity). Wilcoxon signed-rank tests (for paired samples over time) were performed to compare levels of EC markers in cSLE at t=1 and t=2 and lipids at t=1 and t=2. To investigate the potential link between levels of EC markers with capillary patterns, Spearman’s correlation analysis was performed. A p value <0.05 was considered statistically significant. Statistical analysis was performed using IBM SPSS statistics software V.28 and GraphPad Prism V.9 (GraphPad Software). Since this is an exploratory study, we did not perform multiple testing correction.

## Results

### Demographic profile of study subjects

47 patients with cSLE (of which 30 were treatment naïve) and 42 HCs had available samples for inclusion in cross-sectional analyses. Median age at diagnosis in cSLE was 15 years (IQR 12–16 years). At t=1, median SLEDAI was 13 (IQR 9–20) for treatment-naïve patients and median SLEDAI was 8 (IQR 2.5–13.5) for non-naïve patients. Median disease duration at t=1 was 0 month (IQR 0–18 months) for treatment-naïv†e patients and 42 months (IQR 13.6–62.5) for non-naïve patients (n=17). All demographic and clinical characteristics of patients with SLE and (age and sex comparable) HC are depicted in table 1.

**Table 1:** Demographics and clinical characteristics of patients with cSLE and HC

	Patientsn=47	HCn=42	P value*
Female, n (%)	40 (85.1)	36 (85.7)	0.935
Treatment naïve, n (%)	30 (63.8)		
Race/ethnicity, n (%)			<0.001
Caucasian	20 (42.6)	37 (88)	
African/Afro-Caribbean	17 (36.2)	2 (4.8)	
North-African/Middle-Eastern	4 (8.5)	2 (4.8)	
Mixed/other	4 (8.5)	1 (2.5)	
Asian	2 (4.3)	0 (0)	
Age at diagnosis, median (IQR)	15 (12–16)	15 (12–16)†	0.276
BMI at t=1, median (IQR)	19.8(17.3–22.9)		
SLEDAI at diagnosis, median (IQR)	14 (10–18.5)		
Paired samples (n=31 patients with cSLE)			
SLEDAI at t=1 (IQR)	12 (6–18)		
SLEDAI at t=2 (IQR)	2 (1–4)		
Age at sample t=1, years (IQR)	16 (14–17)		
Age at sample t=2, years (IQR)	17 (16–18)		
Disease duration at t=1, months (IQR)	0 (0–18)		
Disease duration at t=2, months (IQR)	16.5 (10–53)		
Capillaroscopy pattern (n=32 patients with cSLE)			
Normal, n (%)	4 (12.5)		
Microangiopathy, n (%)	22 (68.8)		
Scleroderma pattern, n (%)	6 (18.8)		
ANA at diagnosis, n (%)	44 (93.6)		
Anti-ds-DNA, n (%)	36 (76.6)		
Anti-RNP, n (%)	22 (46.8)		
Anti-Sm, n (%)	17 (36.2)		
Cutaneous involvement, n (%)	35 (74.5)		
Nephritis, n (%)	22 (46.8)		
Neuropsychiatric involvement, n (%)	7 (14.9)		
Arthritis, n (%)	34 (72.3)		

* Mann -Whitney U test between HC and all patients with cSLE (n=47).

† Age at date of MRI/blood sampling.

Anti-ds-DNAanti-double stranded DNAAnti-RNPantinuclear ribonucleoproteinAnti-Smanti-SmithBMIbody mass indexcSLEchildhood-onset SLEHChealthy controlSLEDAISystemic Lupus Erythematosus Disease Activity Index

31/47 patients could be included in the longitudinal analyses with active disease at t=1 and low disease activity at t=2. In n=11 patients we did not have second samples (yet). Five excluded patients had low disease activity (SLEDAI≤4) at t=1 and active disease at t=2 (median SLEDAI 8 (range 6–33)). Five patients had minimal active disease (SLEDAI=4) at t=1 and SLEDAI <4 at t=2, these patients were included for longitudinal analyses.

### Serum EC marker levels in patients with cSLE versus HC at t=1

**Figure 1:**
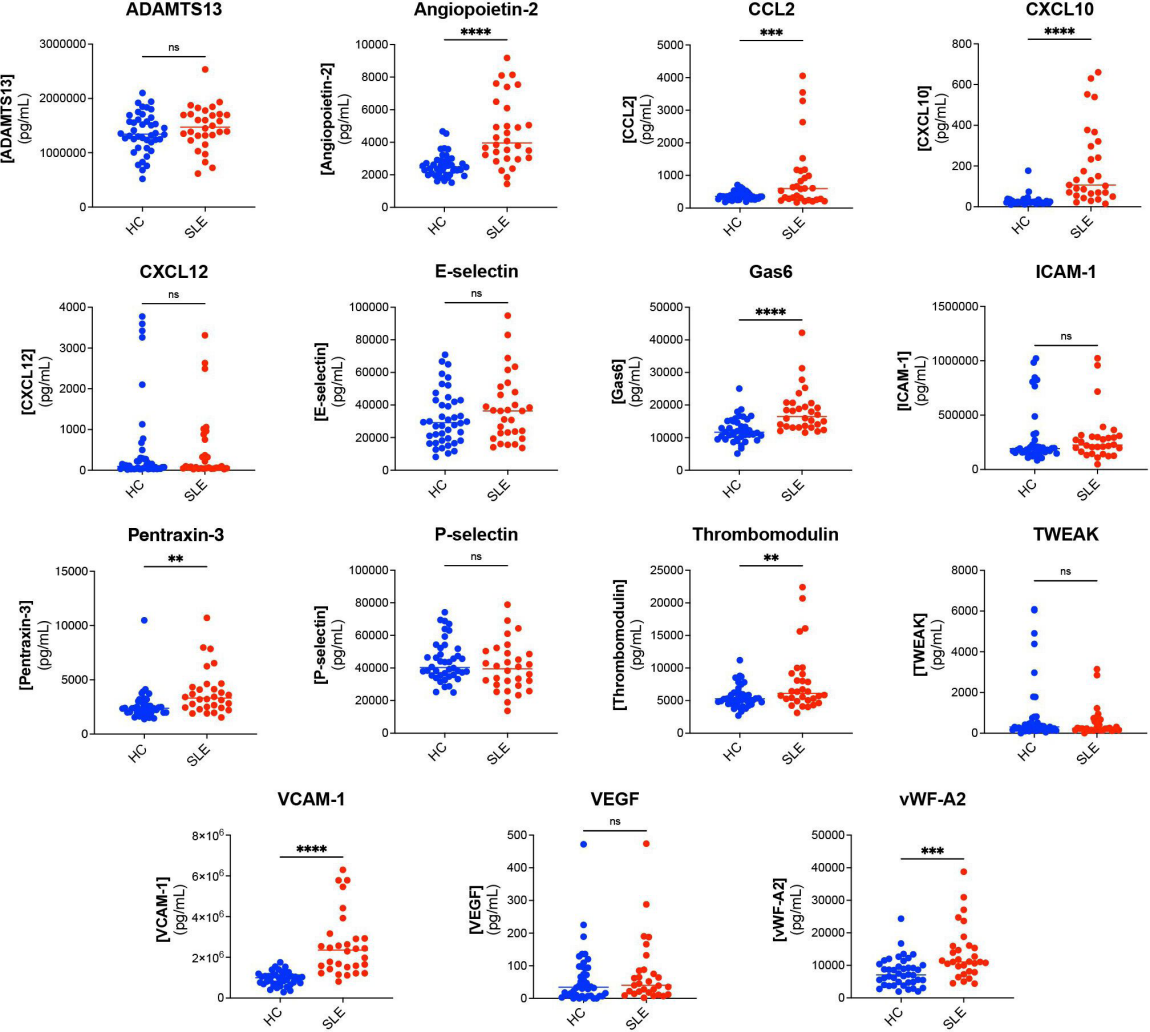
Serum EC marker levels in childhood-onset SLE (cSLE) at t=1 compared with HC. Serum concentration (pg/mL) of each EC marker for all patients with cSLE at t=1 (n=47) compared with HC (n=42). The horizontal line depicts the median serum concentration (pg/mL) of each EC marker for HC and all patients with cSLE. **P<0.01, ***p<0.001, ****p<0.0001, as calculated by Mann-Whitney U test. ADAMTS13, a disintegrin-like and metalloprotease with thrombospondin type 1 motif; CCL2, chemokine (C-C motif) ligand 2; CXCL10, C-X-C motif chemokine ligand 10; CXCL12, C-X-C motif chemokine ligand 12; EC, endothelial cell; Gas6, growth arrest-specific gene 6; HC, healthy control; ICAM-1, intercellular adhesion molecule 1; ns, non-significant; TWEAK, tumour necrosis factor (TNF)-like weak inducer of apoptosis, VCAM-1, vascular cell adhesion molecule 1; VEGF, vascular endothelial growth factor; vWF, von Willebrand factor.

Serum levels of angiopoietin-2, CCL2, CXCL10, GAS6, pentraxin-3, thrombomodulin, VCAM-1 and vWF-A2 were significantly higher in all patients with cSLE (n=36/47 with active disease) compared with HC (n=42). Serum levels of ADAMTS13, CXCL12, E-selectin, ICAM-1, P-selectin, TWEAK and VEGF did not differ between the groups (figure 1 and online supplemental table S1). When evaluating these EC markers in only treatment-naïve patients with cSLE (n=30) and compared with HC, similar findings were obtained (online supplemental figure S1).

### EC markers in patients with cSLE in different disease activity states versus HC at t=1

**Figure 2:**
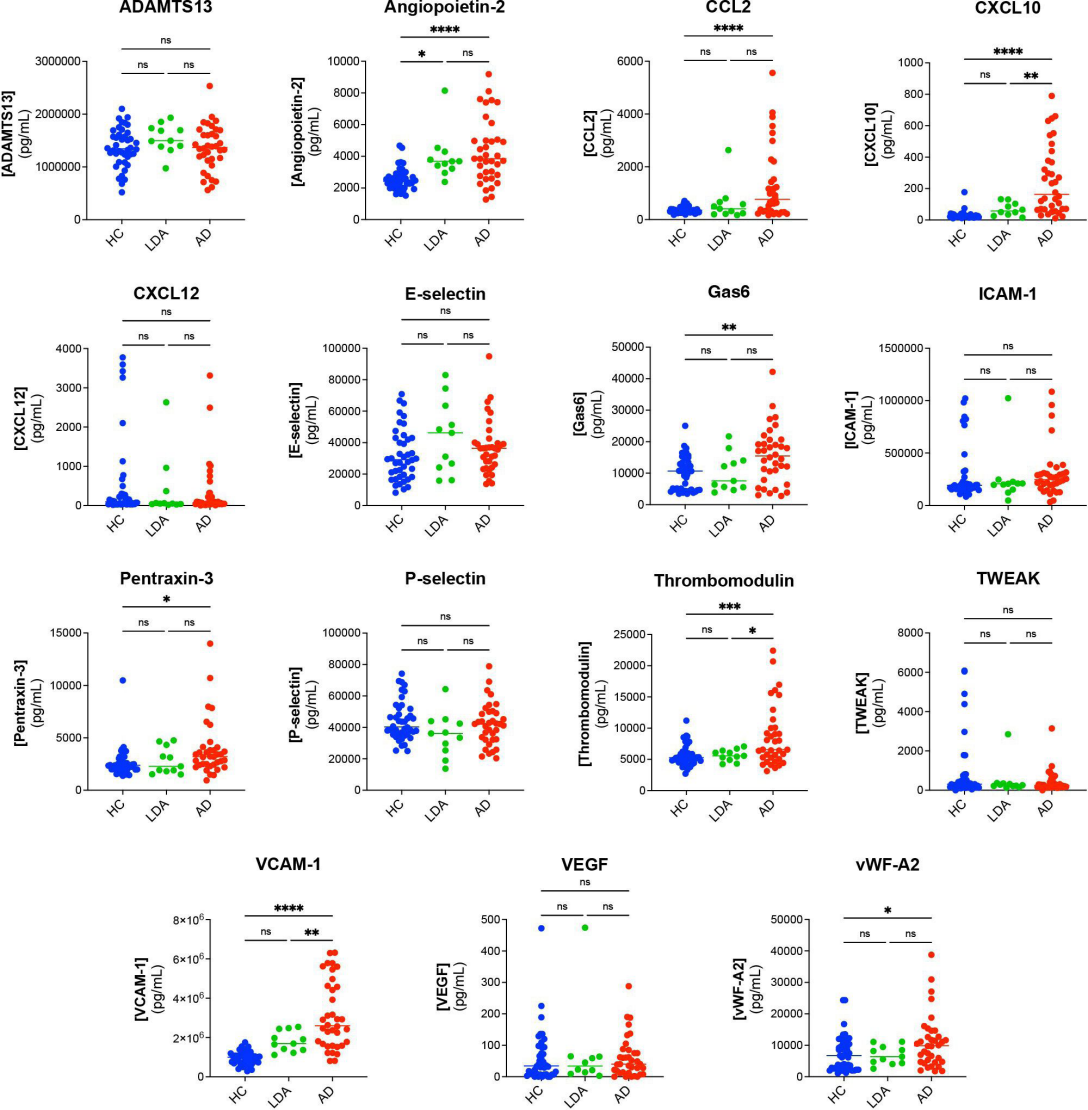
Comparing serum EC marker levels from HC with patients with childhood-onset SLE (cSLE) at t=1 in low disease activity (SLEDAI≤4 in n=11) and in active disease (SLEDAI>4 in n=36). The horizontal line depicts the median serum concentration (pg/mL) of each EC marker. *P<0.05, **p<0.01, ***p<0.001, ****p<0.0001, as calculated by Mann-Whitney U test. AD, active disease; ADAMTS13, a disintegrin-like and metalloprotease with thrombospondin type 1 motif; CCL2, chemokine (C-C motif) ligand 2; CXCL10, C-X-C motif chemokine ligand 10; CXCL12, C-X-C motif chemokine ligand 12; EC, endothelial cell; Gas6, growth arrest-specific gene 6; HC, healthy control; ICAM-1, intercellular adhesion molecule 1; LDA, low disease activity; ns, non-significant; SLEDAI, Systemic Lupus Erythematosus Disease Activity Index; TWEAK, tumour necrosis factor (TNF)-like weak inducer of apoptosis; VCAM-1, vascular cell adhesion molecule 1; VEGF, vascular endothelial growth factor; vWF, von Willebrand factor.

At t=1, serum levels of CXCL10 (p=0.01), thrombomodulin (p=0.03) and VCAM-1 (p=0.01) were higher in patients with active disease (n=36/47) as compared with those with low disease activity (n=11/47) (figure 2) in cross-sectional analysis. Angiopoietin-2, CCL2, CXCL10, GAS6, pentraxin-3, thrombomodulin, VCAM-1 and vWF-A2 differed between patients with active disease and HC. Additionally, from all EC markers only angiopoietin-2 was higher when comparing low disease activity with HC.

### Correlations of EC marker serum levels with SLEDAI at t=1

Angiopoietin-2, CCL2 and VCAM-1 levels did not correlate with SLEDAI at t=1 (table 2) despite higher median serum concentrations in cSLE versus HC (figure 1). A moderate relation for thrombomodulin (r=0.414, p=0.004) with SLEDAI at t=1 was identified (table 2). A weak correlation for GAS6 (r=324, p=0.003) and weak correlations for P-selectin (r=0.348, p=0.02), pentraxin-3 (r=0.290, p=0.01), CXCL10 (r=0.394, p=0.007), VCAM-1 (r=0.317, p=0.03) and CCL2 (r=0.293, p=0.04) with SLEDAI were found.

**Table 2:** Spearman’s R correlations (r) between EC marker levels and SLEDAI at t=1

	Spearman’s R	P value
ADAMTS13	−0.048	0.75
Angiopoietin-2	0.175	0.24
CCL2	**0.293**	**0.04**
CXCL10	**0.394**	**0.007**
CXCL12	0.008	0.96
E-selectin	−0.213	0.15
GAS6	**0.324**	**0.03**
ICAM-1	0.074	0.62
Pentraxin-3	**0.290**	**0.04**
P-selectin	**0.348**	**0.02**
Thrombomodulin	**0.414**	**0.004**
VCAM-1	**0.317**	**0.03**
TWEAK	−0.001	0.99
VEGF	−0.011	0.94
vWF-A2	0.264	0.08

Bold values represent statistic significant Spearman’s R values.

ADAMTS13a disintegrin-like and metalloprotease with thrombospondin type 1 motifCCL2chemokine (C-C motif) ligand 2CXCL10C-X-C motif chemokine ligand 10CXCL12C-X-C motif chemokine ligand 12ECendothelial cellGAS6growth arrest-specific gene 6ICAM-1intercellular adhesion molecule 1SLEDAISystemic Lupus Erythematosus Disease Activity IndexTWEAKtumour necrosis factor (TNF)-like weak inducer of apoptosisVCAM-1vascular cell adhesion molecule 1VEGFvascular endothelial growth factorvWFvon Willebrand factor

### Correlations between different EC markers

Correlations between all measured EC markers at t=1 are depicted in online supplemental table S2. Strong correlations at t=1 were found for thrombomodulin and GAS6 (r=0.712, p<0.001) and for CXCL12 and TWEAK (r=0.823, p<0.001). These markers are involved in different endothelial functions: EC activation, disturbed angiogenesis and vasculogenesis, as well as in coagulopathy.^33^ There were moderate correlations for angiopoietin-2 and pentraxin-3 (r=0.512, p<0.001), pentraxin-3 and thrombomodulin (r=0.562, p<0.001), CCL2 and CXCL10 (r=0.574, p<0.001), CXCL12 and VEGF (r=0.592, p<0.001), vWF-A2 and GAS6 (r=0.667, p<0.001) and VEGF and TWEAK (r=0.680, p<0.001).

At t=2, correlations between angiopoietin-2 with thrombomodulin/GAS6 changed from weak to moderate. A moderate correlation was observed for VCAM-1 and E-selectin (r=0.507, p=0.002) (online supplemental table S3).

### Longitudinal analyses of EC markers in cSLE

**Figure 3:**
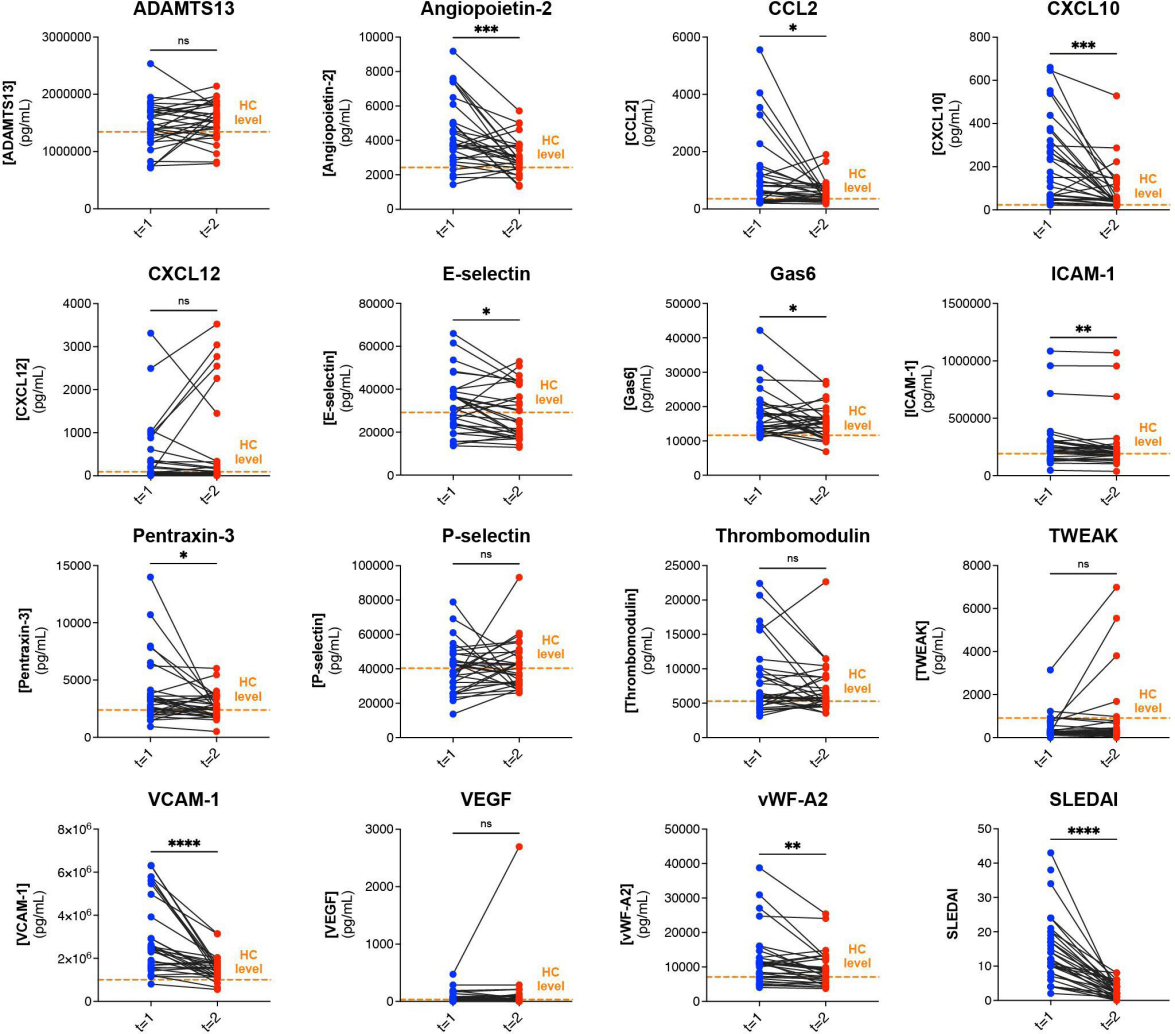
Changes over time in serum EC marker levels and SLEDAI in longitudinal childhood-onset SLE (cSLE) cohort. Serum marker concentrations (pg/mL) and SLEDAI for t=1 (active disease) and t=2 samples (low disease activity) of patients with cSLE (n=31). Orange line indicates the median serum marker concentration for each EC marker in our HC cohort. *P<0.05, **p<0.01, ***p<0.001, ****p<0.0001, as calculated by Wilcoxon signed-rank test. ADAMTS13, a disintegrin-like and metalloprotease with thrombospondin type 1 motif; CCL2, chemokine (C-C motif) ligand 2; CXCL10, C-X-C motif chemokine ligand 10; CXCL12, C-X-C motif chemokine ligand 12; EC, endothelial cell; Gas6, growth arrest-specific gene 6; HC, healthy control; ICAM-1, intercellular adhesion molecule 1; ns, non-significant; SLEDAI, Systemic Lupus Erythematosus Disease Activity Index; TWEAK, tumour necrosis factor (TNF)-like weak inducer of apoptosis; VCAM-1, vascular cell adhesion molecule 1; VEGF, vascular endothelial growth factor; vWF, von Willebrand factor.

Only patients with paired samples that had active disease at t=1 and low disease activity at t=2 (n=31/47) were included for longitudinal analyses. Median time between the blood samples at t=1 and t=2 was 14.5 months (IQR 9–24 months). The median SLEDAI at t=1 was 12 (IQR 6–18) and median SLEDAI at t=2 was 2 (IQR 1–4) (p=0.0004) (figure 3). Serum levels of CXCL10, angiopoietin-2, pentraxin-3, E-selectin, CCL2, VCAM-1 and vWF-A2 decreased over time (p=0.005, p=0.005, p=0.03, p=0.03, p=0.02, p=0.0004 and p=0.0007, respectively), while serum levels of TWEAK increased (p=0.04). At t=2, many elevated EC markers after treatment were not different from those in HC. However, several markers remained significantly increased compared with HC: angiopoietin-2, CCL2, CXCL10, GAS6, thrombomodulin and VCAM-1 (table 3). The outliers in the TWEAK, VEGF and CXCL12 levels at t=2 did not correlate with SLEDAI.

**Table 3:** Mean serum EC levels for HC versus cSLE (all, LDA or AD) at t=2

	HCn=**42**	cSLE, t=2					
		All patients (n=36)	P value	LDA (n=31)	P value	AD (n=5)	P value
ADAMTS13	1 344 200 (1 167 727–1 618 825)	1 399 650 (1 169 950–1 696 350)	ns	1 496 900 (1 385 600–1 733 950)	0.011	1 367 650 (1 128 725–1 677 775)	ns
Angiopoietin-2	2429 (2068–2857)	3715 (2950–4967)	**0.003**	3683 (3219–4293)	ns	3845 (2774–5037)	**<0.001**
CCL2	356 (288–455)	638 (282–1233)	**0.001**	410 (217–666)	ns	769 (311–1501)	ns
CXCL10	23 (20–28)	125 (54–303)	**<0.001**	58 (32–110)	0.017	163 (68–375)	**0.014**
CXCL12	94 (35–294)	70 (33–309)	ns	51 (36–368)	ns	67 (38–295)	ns
E-selectin	29 170 (18 164–42 955)	36 353 (24 220–47 929)	ns	46 222 (23 220–63 471)	ns	36 353 (24 631–39 901)	ns
GAS6	11 657 (10 124–15 096)	15 957 (13 093–19 422)	**0.002**	7530 (5541–14 019)	<0.001	15 453 (8524–20 266)	**0.010**
ICAM-1	191 812 (156 443–238 270)	226 510 (1 693 340–301 594)	ns	206 851 (152 322–226 511)	ns	246 580 (170 911–307 649)	ns
Pentraxin-3	2389 (1983–2883)	3165 (2202–4100)	ns	2277 (1809–4333)	ns	3210 (2422–4031)	ns
P-selectin	40 308 (35 492–51 894)	41 217 (32 057–48 543)	ns	36 145 (25 351–43 991)	ns	42 166 (32 188–49 752)	ns
Thrombomodulin	5275 (4517–6135)	6399 (5049–9070)	**0.028**	5564 (4912–6399)	ns	6556 (5103–10 058)	**<0.001**
VCAM-1	1 006 903 (701 340–1 155 362)	2 421 200 (1 579 300–3 924 350)	**0.001**	1 689 750 (1 365 500–2 437 350)	ns	2 599 925 (1 608 063–4 613 425)	ns
TWEAK	307 (165–574)	258 (177–380)	ns	258 (214–347)	ns	245 (170–533)	ns
VEGF	35 (8–95)	40 (14–75)	ns	34 (13–64)	ns	40 (13–83)	ns
vWF-A2	7102 (4576–10 147)	10 790 (7276–13 918)	ns	6388 (4316–9463)	0.035	9873 (4721–14 162)	ns

Median serum levels (pg/mL) (IQR). Mann -Whitney U test for cSLE versus HC.

P values are given for Mann-Whitney U test between HC and all patients, HC and LDA patients, and HC and AD patients.

Bold values represent significant differences in mean serum EC levels for HC versus cSLE.

ADactive diseaseADAMTS13a disintegrin-like and metalloprotease with thrombospondin type 1 motifCCL2chemokine (C-C motif) ligand 2cSLEchildhood-onset SLECXCL10C-X-C motif chemokine ligand 10CXCL12C-X-C motif chemokine ligand 12ECendothelial cellGAS6growth arrest-specific gene 6HChealthy controlICAM-1intercellular adhesion molecule 1LDAlow disease activitynsnon-significantTWEAKtumour necrosis factor (TNF)-like weak inducer of apoptosisVCAM-1vascular cell adhesion molecule 1VEGFvascular endothelial growth factorvWFvon Willebrand factor

### Correlation of EC marker serum levels with nailfold capillary pattern

In 32/47 patients with cSLE, nailfold capillaroscopy data were available. No significant correlations by Spearman’s rank test were found between type of capillary pattern and EC marker levels at t=1 (n=32 cSLE), but a weak positive correlation was found between levels of angiopoietin-2 with a scleroderma capillary pattern at t=2 (n=6 cSLE) (R^2^=0.167, *F*(2, 25)=2514, p=0.039) (data not shown).

### Lipids in cSLE and HC

Lipids could be measured in a subset of patients with cSLE (n=33/47) and in all HCs (n=42). Although median levels of TG, ApoB and ApoB/ApoA1 ratios were increased in patients with cSLE at t=1 (n=28/33 with active disease) compared with HC, values were still within the physiological ranges for these lipids (online supplemental table S4). Median levels of ApoA1 and HDL were markedly decreased in cSLE, which persisted at t=2 (n=20/22 with low disease activity), but these values were also still within normal physiological range. Weak correlations were observed between TG (r=0.452, p=0.008), ApoB/ApoA1 (r=0.405, p=0.020), ApoB (r=0.483, p=0.004) and SLEDAI.

When analysing correlations between lipids and EC markers, moderate correlations were found between HDL and VCAM-1, TG and thrombomodulin, ApoA1 and VCAM-1, ApoB and thrombomodulin and between ApoB/ApoA1 and pentraxin-3 (table 4). After treatment at t=2, differences in lipids between patients with cSLE and HC disappeared over time.

**Table 4:** Correlations between EC markers and lipids at t=1

	Total cholesterol (mmol/L)	HDL(mmol/L)	LDL (mmol/L)	Triglycerides (mmol/L)	ApoA1 (mg/dL)	ApoB (mg/dL)	ApoB/ApoA1 ratio (mg/dL)
CXCL10		r=−0.518		r=0.474	r=−0.410		r=0.306
ADAMTS13				r=0.262			
Angiopoietin-2		r=−0.567		r=0.381	r=−0.512		r=0.363
Pentraxin-3		r=−0.455		r=0.288	r=−0.472	r=0.314	r=0.523
E-selectin					r=−0.331		
Thrombomodulin				r=0.437			r=0.332
P-selectin	r=0.317					r=0.347	r=0.243
CCL2				r=0.341			r=0.345
VCAM-1		r=−0.607		r=0.404	r=−0.596		r=0.351
ICAM-1							
vWF-A2				r=0.301		r=0.301	r=0.371
GAS6		r=−0.411			r=−0.392		r=0.352
CXCL12							
TWEAK							
VEGF							

Spearman’s rank correlation reported for correlations between 15 EC markers and lipids. Only significant correlations are shown.

Light grey: weak correlations (r=0.3–0.5). Darker grey: moderate correlations (r=0.5–0.7).

ADAMTS13a disintegrin-like and metalloprotease with thrombospondin type 1 motifApoA1apolipoprotein AIApoBapolipoprotein BCCL2chemokine (C-C motif) ligand 2CXCL10C-X-C motif chemokine ligand 10CXCL12C-X-C motif chemokine ligand 12ECendothelial cellGAS6growth arrest-specific gene 6HDLhigh-density lipoproteinICAM-1intercellular adhesion molecule 1LDLlow-density lipoproteinTWEAKtumour necrosis factor (TNF)-like weak inducer of apoptosisVCAM-1vascular cell adhesion molecule 1VEGFvascular endothelial growth factorvWFvon Willebrand factor

**Figure 4:**
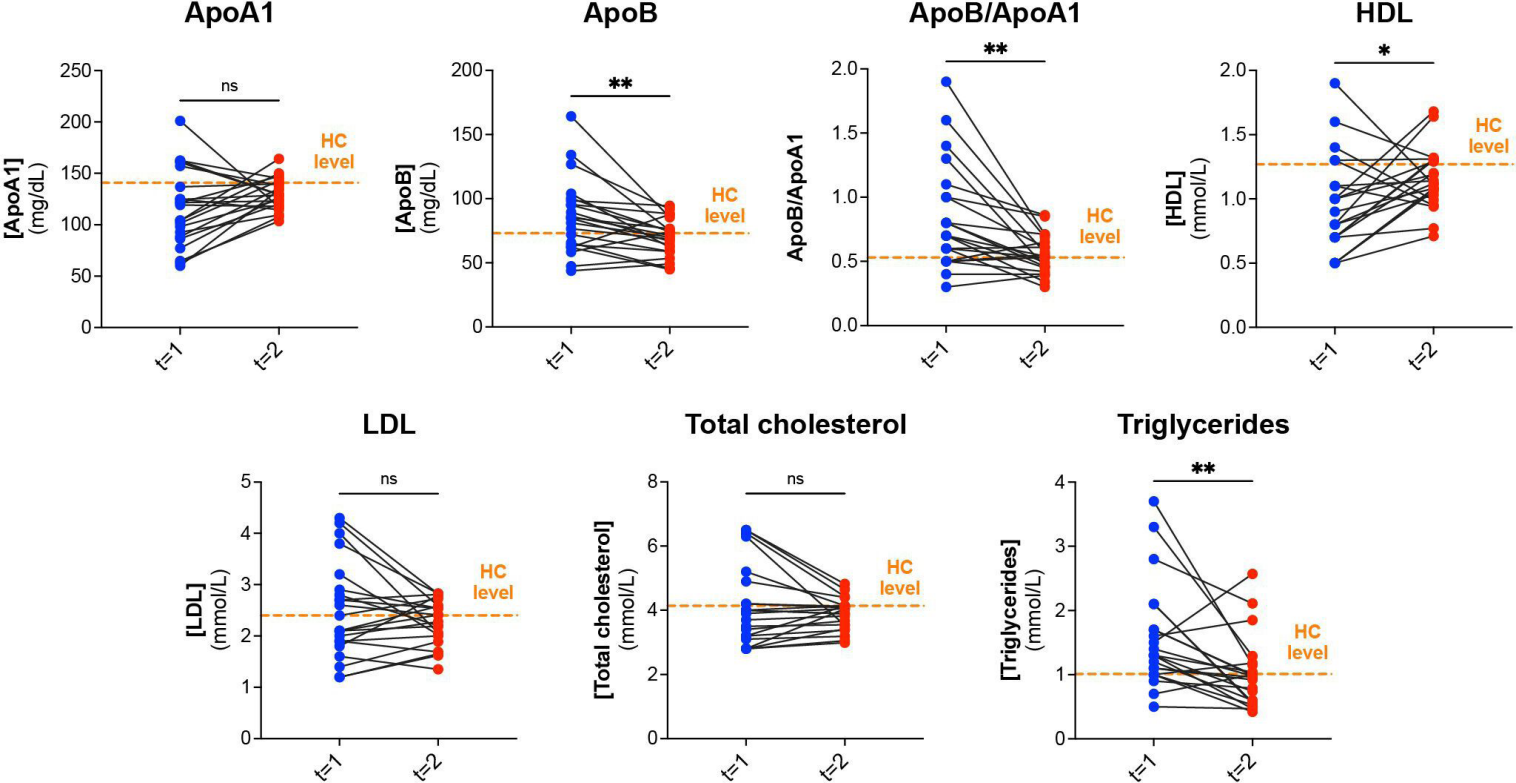
Longitudinal measurements of lipids (mg/dL or mM) at t=1 (active disease) and t=2 (low disease activity) in childhood-onset SLE (cSLE; n=20). On the x-axis, blue dots represent t=1, red dots t=2. Serum levels of lipids are displayed on the y-axis. Orange line indicates median concentrations for lipids in our HC cohort. *P<0.05, **p<0.01, as calculated by Wilcoxon signed-rank test. ApoA1, apolipoprotein AI; ApoB, apolipoprotein B; HC, healthy control; HDL, high-density lipoprotein; LDL, low-density lipoproteinns; ns, non-significant.

Longitudinal measurements of lipids (with high disease activity at t=1 and low disease activity at t=2) were only possible in a subgroup of patients with cSLE (n=20/33). ApoB (p=0.0032) and ApoB/ApoA1 ratio (p=0.0014) and TG (p=0.0042) decreased over time, while HDL levels (p=0.03) increased. Total cholesterol, LDL and ApoA1 levels did not differ over time (p=0.21, p=0.19 and p=0.085, respectively) (figure 4).

## Discussion

Our study shows that many of the assessed SLE-associated markers involved in EC function were upregulated in patients with cSLE compared with HC, especially in treatment-naïve and active disease. Angiopoietin-2, CCL2 and VCAM-1 were upregulated in patients with cSLE compared with HC but interestingly also in some patients with lower disease activity (at t=1). Despite low median disease activity, angiopoietin-2, CCL2, CXCL10, GAS6, thrombomodulin and VCAM-1 remained significantly upregulated in patients with cSLE compared with HC (at t=2). This implies that the endothelium in (a subset of) patients with cSLE remains in a chronically active state, regardless of disease activity. The aforementioned markers are involved in a broad spectrum of biological functions of EC, including vascular inflammation, EC activation and a proangiogenic state. HDL, TG, ApoA1 and ApoB/ApoA1 were dysregulated in patients with cSLE compared with HC (at t=1), but still within the physiological range. In longitudinal analyses, differences in lipids between patients with cSLE and HC disappeared over time, which is probably related to lower disease activity (and treatment).

Thrombomodulin is found on the surface of vascular EC and acts as a receptor for thrombin.^34^ Studies indicate that serum thrombomodulin is released when ECs are damaged.^35 36^ Dysfunction in either the amount or quality of thrombomodulin may contribute to thrombogenesis, which commonly occurs in patients with SLE.^37^ Importantly, thrombomodulin is being considered as a marker for EC damage and has been linked to active vasculitis in SLE.^35^ In line with our results, Lee *et al* demonstrated upregulated levels of thrombomodulin in cSLE compared with HC as well as a significant relation between thrombomodulin and SLEDAI.^38^ This correlation between thrombomodulin and SLEDAI was also seen in an aSLE cohort.^39^

VCAM-1 is a cell surface adhesion molecule, overexpressed on EC that plays a role in the immune response. In activated EC, VCAM-1 contributes to the adhesion and migration of immune cells from the blood to sites of inflammation. We found VCAM-1 to be upregulated in cSLE but we did not find a correlation between VCAM-1 levels and disease activity. In line with our findings, a study in aSLE reported upregulated serum levels of VCAM-1 but they found a weak correlation for VCAM-1 with disease activity.^35^

CCL2 (also known as MCP-1) is a chemokine that is produced by various cell types, for example, macrophages, fibroblasts and ECs, in response to inflammation. When there is vascular injury or inflammation, such as in atherosclerosis or other inflammatory conditions as, for example, SLE, ECs lining the blood vessels can produce CCL2. The locally produced CCL2 acts as a signalling molecule. It binds to its receptor (CCR2) on the surface of monocytes. This binding triggers a series of events that result in the monocytes leaving the bloodstream, adhering to the endothelium and migrating through the blood vessel wall into the subendothelial space.^40^ A study with adult patients with SLE^41^ demonstrated an upregulation of CCL2 serum levels in SLE compared with HC, without correlation with disease activity, which is in accordance with our results. These findings suggest a dysregulated endothelium *irrespective* of SLE disease activity.

CXCL10 is released by a diverse range of cells, including leucocytes, activated neutrophils and ECs. CXCL10 attracts activated Th1 lymphocytes, monocytes and natural killer cells to the area of inflammation.^42^ It has been shown that CXCL10 is increased in patients with aSLE compared with HC.^43^ In that study, CXCL10 correlated strongly with disease activity. In our study, CXCL10 was also upregulated in cSLE compared with HC and there was a weak correlation with SLEDAI (r=0.39, p=0.008).

The majority of the assessed lipids differed significantly in patients with cSLE compared with HC in our study, despite means of values falling within the normal physiological range. As shown previously in the APPLE (Atherosclerosis Prevention in Pediatric Lupus Erythematosus) study,^44^ mean levels of HDL, LDL and TG were also in the normal or borderline ranges in that cSLE cohort. Another cross-sectional study showed a significant difference in ApoB and TG levels between cSLE and HC.^45^ There was no significant difference in levels of total cholesterol, LDL-C, HDL-C and ApoA1 levels. ApoB/Apo1 ratios were not reported. Interestingly, based on multiserum metabolomics analysis, it has been suggested that high ApoB/ApoA1 ratio could function as a potential biomarker of an increased cardiometabolic risk.^46^ It has been reported that the ApoB/ApoA1 ratio can assist in classifying patients who require increased disease monitoring, lipid modification or lifestyle changes.^46^ In our study, ApoB/ApoA1 ratio was elevated in patients with cSLE (with normal body mass index (BMI) ranges) with active disease compared with HC. However, after treatment, this difference disappeared in low disease activity states. Similarly, most of the differences in lipids between patients with cSLE and HC disappeared. This implies that there was a trend towards dysregulated lipids during active disease with positive effect of anti-inflammatory treatment in these patients. However, due to limited sample volumes we were only able to measure lipids longitudinally in 22/47 patients with cSLE. Interestingly, in a subset analysis within the previously mentioned APPLE study, 36% of patients with cSLE experienced ongoing atherosclerosis, which was not predictable by metabolic biomarkers. This suggests the presence of non-lipid drivers for atherosclerosis, emphasising the importance of considering such factors in the management of these patients.^47^

Recently, we proposed that an abnormal nailfold capillary pattern reflects early vasculopathy in patients with cSLE, since we observed that more than 50% of patients with a capillary scleroderma pattern already had SLE-related disease damage within 5 years after diagnosis.^28^ In the current study, 68.8% of patients with cSLE had a capillary microangiopathy pattern and 18.8% showed a capillary scleroderma pattern at diagnosis. In an exploratory manner, we have analysed possible relationships between EC marker levels and abnormal nailfold capillaroscopic patterns. Angiopoietin-2 showed a weak correlation with a scleroderma pattern. Angiopoeitin-2 was also elevated irrespective of disease activity in our patients with cSLE. This EC marker is involved in the ‘disturbed angiogenesis’ of EC function.^33^ These results are in line with a previous study, in which higher levels of angiopoietin-2 in SLE compared with HC were found. Moreover, there was no correlation between angiopoietin-2 levels and SLEDAI.^48^ In the aforementioned study by Lee *et al*,^38^ angiopoietin-2 was also upregulated in patients with cSLE compared with HC, but did not correlate with SLE disease activity, similar to our current findings. We hypothesise that high angiopoietin-2 levels might reflect the vasculopathy and disturbed angiogenesis that is observed by nailfold capillaroscopy (abnormal nailfold capillaries with capillary giants, haemorrhages and abnormal capillary morphology). To our knowledge, there is only one study that studied the potential correlation between EC markers and NVC patterns.^49^ Angiopoietin-2 levels were not measured in this study, but a correlation between VEGF and microvascular abnormalities in nailfold capillaroscopy was reported. In our study, we did not observe a correlation between VEGF levels and an abnormal capillary pattern. Another study also stated that angiopoietin-2 may be used as a potential biomarker in SLE, since this marker seemed to have potential to differentiate patients with SLE from those with rheumatoid arthritis, osteoarthritis, gout, Sjögren’s syndrome and ankylosing spondylitis.^48^ It is important to mention that in our study not all samples were taken simultaneously with capillaroscopy examination. Although we have shown earlier that most capillary patterns do not change over time,^28^ this is a limitation. Future studies will have to show whether angiopoietin-2, in combination with NVC, might be used as a biomarker for (ongoing) vascular inflammation.

This is the first longitudinal study in cSLE assessing SLE-associated markers involved in EC function in combination with longitudinal measurements of lipids. The number of studies on EC markers in patients with cSLE is very limited. The uniqueness of our cohort also lies in the fact that more than half of the patients (30/47, 63.8%) were treatment naïve at the moment of first blood sample. These treatment-naïve samples reflect an endothelial state that is solely attributable to the disease itself, with no influence from medication.

Nonetheless, there are also several limitations. First, we were not able to perform longitudinal analyses in all patients, if patients did not yet achieve inactive disease. Additionally, we were not able to perform lipid measurements in all patients due to a lack of material in a substantial part of our patients. Therefore, it was only possible to measure the lipids longitudinally in 50% of the cSLE cohort which might have biased our results with less statistical significance. Other limitations are the lack of nailfold capillaroscopy data in a considerable number of patients with cSLE (n=15) and the limited follow-up period (mean 31 months, median 16.5 months) after diagnosis. Therefore, we were not able to determine any effects caused by medication and/or disease duration over time on the measured EC markers and lipids. Moreover, it is of note that HCs were not matched with patients with cSLE. Therefore, ethnic backgrounds of the HC group differed substantially from the cSLE group, with a majority of Caucasian subjects in the HC group (88% compared with 42% in cSLE patient group) which might have an effect on biology and their cardiovascular risks. In addition, BMIs of the HCs were not obtained.

It is important to decrease the overall incidence of CVD-related morbidity and mortality in SLE by using appropriate prevention and treatment strategies. To date, established screening protocols for detecting or monitoring CVD in cSLE do not exist. However, for patients with cSLE who have become adults, such protocols would be valuable, as we know they suffer from higher disease activity, longer disease duration and premature atherosclerosis at a relatively young age, compared with aSLE. There is a pressing and unmet need to develop improved methods to stratify patients with cSLE who are at risk for CVD in order to start preventive treatment. Future studies should therefore further elucidate the changes in EC markers in combination with lipids over time. We urge for more thorough investigations on the relation between the EC dysregulation in SLE and increased risk of premature atherosclerosis and CVD, with an emphasis on the differences in cSLE and adult-onset patients.

## Conclusion

In cSLE, markers of endothelial activation were dysregulated. Some of those EC markers remained dysgregulated in a subset of patients with cSLE despite low disease activity. This study could aid in unravelling a part of the pathophysiology of endothelial dysregulation in patients with cSLE. These results suggest a role for the dysregulated endothelium in early and later phases of cSLE and if so, this could have implications for future screening protocols for vascular health in cSLE.

## supplementary material

10.1136/lupus-2024-001190 online supplemental file 1

## Data Availability

Data are available upon reasonable request.
