# Supplementary material for: Learning from serum markers reflecting endothelial activation: longitudinal data in childhood-onset systemic lupus erythematosus
Source: Lupus Sci Med. 2024 Sep 5;11(2):e001190. doi: 10.1136/lupus-2024-001190 (PMC11381702; doi:10.1136/lupus-2024-001190)
Supplement: online supplemental file 1 [file lupus-11-2-s001.pdf]

Supplementary file

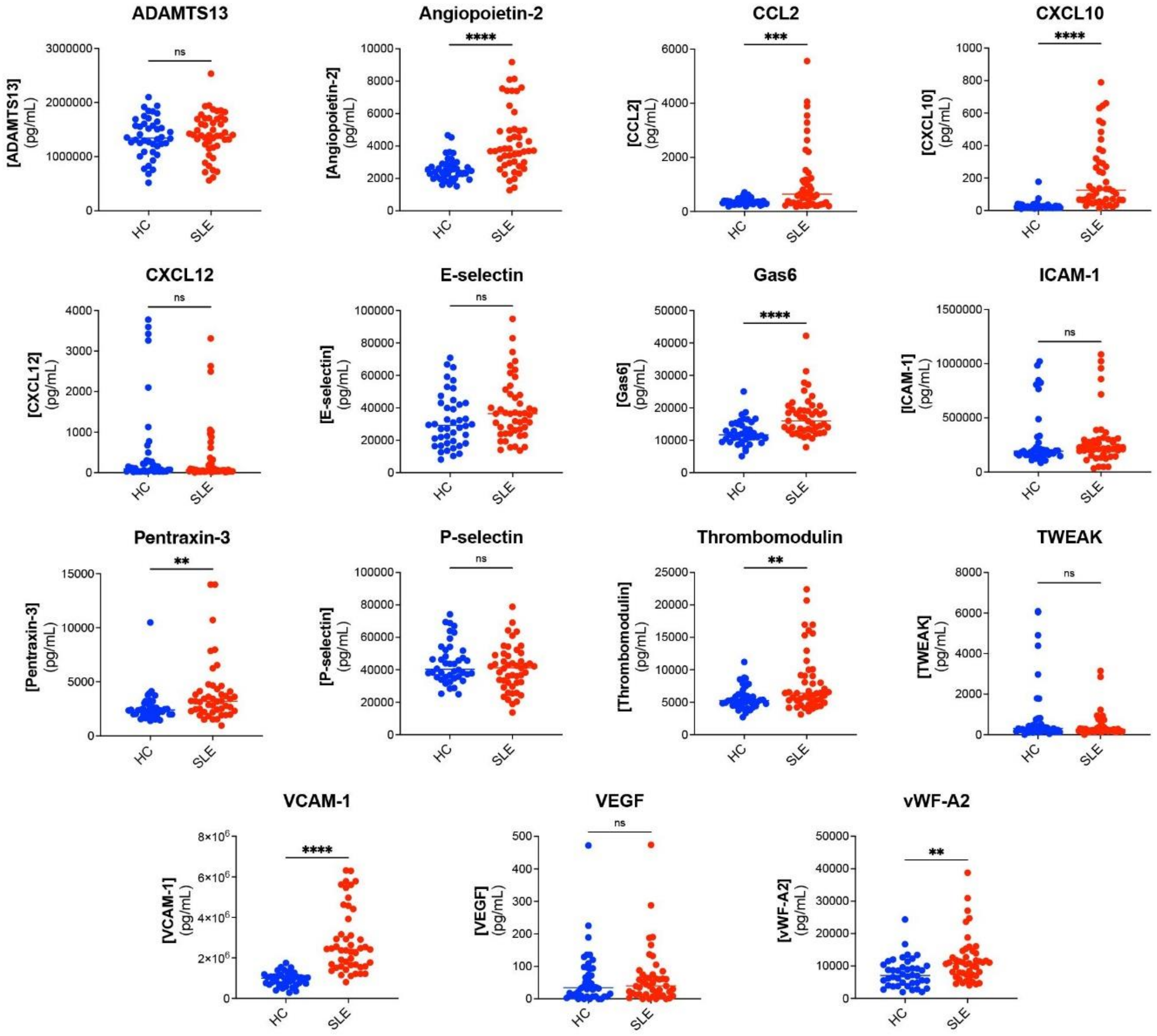

Figure S1. EC marker levels in treatment-naive cSLE versus HC at t=1.

Serum concentration (pg/mL) of each EC marker, the horizonal line depicts the median serum concentration. .

Blue and red dots representing cSLE patients and HC respectively.

*$p < 0.05$, **$p < 0.01$, ***$p < 0.001$, ****$p < 0.0001$, as calculated by Mann Whitney U test.

Abbreviations: ADAMTS13: A Disintegrin-like and Metalloprotease with Thrombospondin Type 1 Motif, CCL2: chemokine (C-C motif) ligand 2, CXCL10: C-X-C Motif Chemokine Ligand 10, CXCL12: C-X-C motif chemokine ligand 12, EC: endothelial cell, GAS6: GAS6: growth arrest-specific gene 6, HC: healthy control, ICAM-1: ICAM-1: Intercellular Adhesion Molecule 1, SLE: systemic lupus erythematosus, SLEDAI: Systemic Lupus Erythematosus Disease Activity Index TWEAK: Tumor necrosis factor (TNF)-like weak inducer of apoptosis, VCAM-1: Vascular Cell Adhesion Molecule 1, VEGF: Vascular Endothelial Growth-Factor, vWF-Von Willebrand Factor

Table S1. Median serum levels of EC markers for HC vs cSLE at t =1.

	HC	cSLE	
	**All HC (n=42)**	**All patients (n=47)**	**p-value**
**ADAMTS13**	1344200 (1167727 – 1618825)	1399650 (1169950 – 1696350)	0.434
**Angiopoietin-2**	2429 (2068 – 2857)	3715 (2950 – 4967)	**0.003**
**CCL2**	356 (288 – 455)	638 (282 – 1233)	**0.001**
**CXCL10**	23 (20 -28)	125 (54 – 303)	**<0.001**
**CXCL12**	94 (35 – 294)	599 ± 1095	0.768
**E-Selectin**	29170 (18164 – 42955)	36353 (24220 – 47929)	0.347
**GAS6**	11657 (10124 – 15096)	15957 (13093 – 19422)	**0.002**
**ICAM-1**	191812 (156443 – 238270)	226510 (1693340 – 301594)	0.223
**Pentraxin-3**	2389 (1983 – 2883)	3165 (2202 – 4100)	0.118
**P-Selectin**	40308 (35492 – 51894)	41217 (32057 – 48543)	0.353
**Thrombomodulin**	5275 (4517 – 6135)	6399 (5049 – 9070)	**0.028**

**VCAM-1**	1006903<br>(701340 – 1155362)	2421200<br>(1579300 – 3924350)	**0.001**
**TWEAK**	307<br>(165 – 574)	258<br>(177 - 380)	0.532
**VEGF**	35<br>(8 – 95)	40<br>(14 – 75)	0.613
**vWF-A2**	7102<br>(4576 – 10147)	10790<br>(7276 – 13918)	0.531

Median serum levels (pg/mL) (IQR). Mann Whitney U-test for cSLE vs HC. NS: Non-significant

Abbreviations: ADAMTS13: A Disintegrin-like and Metalloprotease with Thrombospondin Type 1 Motif, CCL2: chemokine (C-C motif) ligand 2, CXCL10: C-X-C Motif Chemokine Ligand 10, CXCL12: C-X-C motif chemokine ligand 12, EC: endothelial cell, GAS6: GAS6: growth arrest-specific gene 6, HC: healthy control, ICAM-1: ICAM-1: Intercellular Adhesion Molecule 1, SLE: systemic lupus erythematosus, SLEDAI: Systemic Lupus Erythematosus Disease Activity Index TWEAK: Tumor necrosis factor (TNF)-like weak inducer of apoptosis, VCAM-1: Vascular Cell Adhesion Molecule 1, VEGF: Vascular Endothelial Growth-Factor, vWF-Von Willebrand Factor

	CXCL10	A-TS13	Ang-2	PTX-3	E-Sel	Tm	P-Sel	CCL2	VCAM1	ICAM1	vWF-A2	GAS6	CXCL12	TWEAK	VEGF
**CXCL10**	X							0,57	0,47		0,37				
**A-TS13**		X				0,38					0,39	0,49	0,36		
**Ang-2**			X	0,51		0,39					0,35	0,33	0,31		
**PTX-3**			0,51	X		0,56	0,32				0,47	0,42			
**E-sel**					X									0,30	0,31
**Tm**		0,38	0,39	0,56		X					0,47	0,71			
**P-Sel**				0,32			X								
**CCl-2**	0,57							X	0,31						
**VCAM1**	0,47							0,31	X			0,31			
**ICAM1**										X				0,29	0,44
**vWF-A2**	0,37	0,39	0,35	0,47		0,47					X	0,67			
**GAS6**		0,49	0,33	0,42		0,71			0,31		0,67	X	0,37		
**CXCL12**		0,36	0,31									0,37	X	0,82	0,59
**TWEAK**					0,30					0,29			0,82	X	0,68
**VEGF**					0,31					0,44			0,59	0,68	X

Table S2: Correlation matrix different endothelial markers at t=1 (Pearson's r correlations)
Light grey r-values: weak correlations (r = 0.3 – 0.5). Darker grey: moderate correlations (r = 0.5 - 0.7) Darkest grey: strong correlations (r = 0.7 – 0.9)

Abbreviations: Ang-2: angiopoietin-2, A-TS13: A Disintegrin-like and Metalloprotease with Thrombospondin Type 1 Motif, CCL2: chemokine (C-C motif) ligand 2, CXCL10: C-X-C Motif Chemokine Ligand 10, CXCL12: C-X-C motif chemokine ligand 12, E-Sel: E-selectin, GAS6: GAS6: growth arrest-specific gene 6, HC: healthy control, ICAM-1: ICAM-1: Intercellular Adhesion Molecule 1, P-Sel: P-Selectin, PTX-3: pentraxin-3, SLE: systemic lupus erythematosus, TM: Thrombomodulin, TWEAK: Tumor necrosis factor (TNF)-like weak inducer of apoptosis, VCAM-1: Vascular Cell Adhesion Molecule 1, VEGF: Vascular Endothelial Growth-Factor, vWF-Von Willebrand Factor

	CXCL10	A-TS13	Ang-2	PTX-3	E-Sel	Tm	P-Sel	CCL2	VCAM1	ICAM1	vWF-A2	Gas6	CXCL12	TWEAK	VEGF
**CXCL10**	X		0,37					0,36	0,51						
**A-TS13**		X								-0,42					
**Ang-2**	0,37		X		0,37	0,52			0,38			0,55			
**PTX-3**				X			0,37				0,34				
**E-sel**			0,37		X	0,40			0,51			0,83			
**Tm**		0,38	0,52		0,40	X			0,35			0,57			
**P-Sel**				0,37			X	0,49							
**CCl-2**	0,36						0,49	X	0,31						
**VCAM1**	0,51		0,35		0,51	0,35		0,43	X			0,42			
**ICAM1**		-0,42								X					
**vWF-A2**			0,35	0,34							X	0,59			
**Gas6**			0,55			0,57			0,42		0,59	X	0,37		
**CXCL12**													X	0,85	0,61
**TWEAK**													0,85	X	0,37
**VEGF**													0,61	0,37	X

Table S3: Correlation matrix different endothelial markers at t=2 (Pearson's r correlations)
(Colored, but empty boxes were significantly correlated at t=1, but correlation disappeared at t=2)
Boxes without color show new correlations at t=2, that did not exist at t=1
Light grey r-values: weak correlations (r = 0.3 – 0.5). Darker grey: moderate correlations (r = 0.5 - 0.7) Darkest grey: strong correlations (r = 0.7 – 0.9)

Abbreviations: Ang-2: angiopoietin-2, A-TS13: A Disintegrin-like and Metalloprotease with Thrombospondin Type 1 Motif, CCL2: chemokine (C-C motif) ligand 2, CXCL10: C-X-C Motif Chemokine Ligand 10, CXCL12: C-X-C motif chemokine ligand 12, E-Sel: E-selectin, GAS6: GAS6: growth arrest-specific gene 6, HC: healthy control, ICAM-1: ICAM-1: Intercellular Adhesion Molecule 1, P-Sel: P-Selectin, PTX-3: pentraxin-3, SLE: systemic lupus erythematosus, TM: Thrombomodulin, TWEAK: Tumor necrosis factor (TNF)-like weak inducer of apoptosis, VCAM-1: Vascular Cell Adhesion Molecule 1, VEGF: Vascular Endothelial Growth-Factor, vWF-Von Willebrand Factor

Table S4: Lipids in HC, cSLE at t=1 and cSLE at t=2

**Lipids**	**HC (n=42**	**cSLE t=1 (n=33) (p-value)**	**p-value**	**cSLE t=2 (n=22) (p-value)**	**p-value**
Total cholesterol (mmol/L) <5 mmol/L	4.1 (3.7 – 4.6)	3.9 (3.2 – 4.8)	0.818	1.1 (1.0 – 1.3)	**0.045**
HDL (mmol/L) >1 mmol/L	1.3 (1.1 – 1.4)	0.8 (0.7 – 1.1)	**< 0.001**	1.1 (1.0 – 1.3)	**0.047**
LDL (mmol/L) <3 mmol/L	2.4 (2.0 – 2.8)	2.3 (1.8 – 3.0)	0.727	2.27 ± 0.44	0.395
Triglycerides (mmol/L) <2 mmol/L	1 (0.8 -1.2)	1.3 (1.0 – 2.0)	**< 0.001**	2.3 (2.0 – 2.6)	0.989
ApoA1 (mg/dL) 108-225 mg/dL	135 (127 – 150)	111 (89 – 139)	**< 0.001**	129 (118 – 141)	**0.045**
ApoB (mg/dL) 60-141 mg/dL	74 (63 – 84)	85 (62 – 101)	**0.023**	71 (59 – 79)	0.437
ApoB/ApoA1 ratio 0.3-0.8	0.5 (0.4 – 0.6)	0.7 (0.5 – 1,0)	**< 0.001**	0.5 (0.4 – 0.7)	0.610

Median serum levels (pg/mL) (IQR). Mann Whitney U-test for cSLE vs HC. NS: Non-significant

P-values are given for Mann Whitney U between HC and cSLE t=1 and for HC compared to cSLE t=2.

Abbreviations: ApoA1: Apolipoprotein AI, ApoB: Apolipoprotein B, HC: healthy controls, HDL: high density lipoprotein, LDL: low density lipoprotein, cSLE: childhood-onset systemic lupus erythematosus
